# Association between dietary inflammatory index and atherosclerosis cardiovascular disease in U.S. adults

**DOI:** 10.3389/fnut.2022.1044329

**Published:** 2023-01-05

**Authors:** Jie Zhang, Jundi Jia, Runmin Lai, Xinyi Wang, Xuanye Chen, Wende Tian, Qiyu Liu, Jingen Li, Jianqing Ju, Hao Xu

**Affiliations:** ^1^National Clinical Research Center for Chinese Medicine Cardiology, Xiyuan Hospital, China Academy of Chinese Medical Sciences, Beijing, China; ^2^Graduate School, Beijing University of Chinese Medicine, Beijing, China; ^3^Graduate School, China Academy of Chinese Medical Sciences, Beijing, China; ^4^Department of Cardiovascular Medicine, Dongzhimen Hospital, Beijing University of Chinese Medicine, Beijing, China

**Keywords:** dietary inflammatory index, atherosclerotic cardiovascular disease, NHANES, diet, inflammation

## Abstract

**Objective:**

To evaluate the association between dietary inflammatory index (DII) and Atherosclerotic cardiovascular disease (ASCVD) among U.S. adults.

**Methods:**

We collected data from National Health and Nutrition Examination Survey (NHANES) between 1999 and 2018. Adults who reported complete information to diagnose ASCVD and calculate DII were included. We used three models to differentially adjust the covariates, including age, sex, race or ethnicity, education level, smoking status, poverty, insurance, body mass index, hyperlipemia, hypertension, and diabetes. Logistic regression was used to estimate the Odds Ratio (OR) and 95% confidence interval (95% CI) for ASCVD grouped by DII deciles. We additionally conducted spline smoothing with the generalized additive model (GAM) and the log-likelihood ratio to examine the non-linear relationship between DII and ASCVD. If exists, the segmented linear regression will be used to detect the cutoff point. The subgroup analyses were stratified by various atherosclerotic cardiovascular diseases (i.e., CHD, angina, heart attack, and stroke) and sex.

**Results:**

A total of 48,733 participants (mean age, 47.13 ± 0.19 years) with 51.91% women were enrolled, of which 5,011 were diagnosed with ASCVD. In the crude model, participants in the five highest deciles (D6, 7, 8, 9, and 10) of DII score had a significantly higher risk of having ASCVD compared to those in the first decile. In the fully adjusted model, those in the tenth decile [OR = 1.47, 95% CI = (1.18,1.84)] of DII had a significantly increased risk of ASCVD compared to the first decile. Notably, when DII is above 3, the ASCVD risk increased by 41% for each one increase in DII [OR = 1.41, 95% CI = (1.15,1.73)]. This relationship was more pronounced in females.

**Conclusion:**

Our study revealed a positive and non-linearly association between DII and ASCVD in U.S. adults. This relationship was more pronounced in females. The findings provide a reference for future research and diet recommendations.

## 1. Introduction

Atherosclerotic cardiovascular disease (ASCVD) remains to be the leading cause of morbidity and mortality despite tremendous advances in traditional risk factor management. Therefore, great effort has been put into identifying and managing residual risk factors (1, 2). Growing evidence suggests that inflammation is a novel and effective target to reduce residual risk factors for ASCVD (3, 4). However, since interference with inflammatory pathways can compromise host defenses, optimizing the net benefit remains a challenge (4). Only canakinumab (5) and colchicine (6–8) have been validated to treat ASCVD by targeting inflammation without unacceptable side effects.

Diet is a critical modifiable target for ASCVD prevention and management (9) and the fundamental mechanism has been associated with potential pro- or anti-inflammatory properties of the dietary pattern or dietary components (10–12). Pro-inflammatory foods include processed meats, refined starches, and foods and beverages with added sugars. In contrast, anti-inflammatory foods include dark vegetables, whole grains, fruits, tea, coffee, and wine (11). Obviously, the inflammatory properties of individual foods are not adequate to assess the inflammatory level of various dietary patterns. To quantify the underlying inflammatory level of various diet patterns, in 2009 and 2014, Hébert and colleagues developed and improved the dietary inflammatory index (DII), a literature-derived and population-based scoring system, (13, 14), which has been validated in many studies with a variety of inflammatory markers (10).

Accumulating evidence from diverse populations suggested a positive association between DII and cardiovascular diseases (CVD), such as a 10-year risk for ASCVD, CVD factors, cardiovascular mortality, and CVD risk burden (15–20). That is, the higher the dietary inflammation, the more harmful to people. A secondary analysis of the PREDIMED (Prevención con Dieta Mediterránea) study (21) prospectively examined the relationship between DII and the incidence of CVD (i.e., myocardial infarction, stroke, or cardiovascular death), including 7,169 high-risk participants with a median follow-up of 4.8 years. The results show a direct prospective association between increased diet-associated inflammation and the risk of CVD. As a continuous variable, the HR for each additional standard deviation of the DII was 1.22 (95% CI = 1.06–1.40). In fact, some pro-inflammatory components such as vitamin B12, energy, protein, cholesterol, and carbohydrates are essential to sustain life (14). Some essential ingredients have a non-linear effect on health (22) and DII is non-linear associated with some biomarkers and diseases (23–25). Maybe there is a threshold between DII scores and ASCVD, below which could be considered safe, rather than less is healthier. However, whether a cutoff point exists and its value remains unclear. Furthermore, the association between DII and ASCVD in U.S. adults is still unclear, and whether this relationship persists across genders and various atherosclerotic cardiovascular diseases is controversial (26, 27).

The NHANES^1^ is an ongoing cross-sectional study aiming to reflect the health of the U.S. population (28). Here we aimed to use the NHANES database (1999–2018) to assess the association between DII and ASCVD, identified the cutoff point if exits, and detected the subgroups population most likely to benefit from controlling DII.

## 2. Materials and methods

This cross-sectional study was conducted and reported following the Strengthening the Reporting of Observational Studies in Epidemiology (STROBE) Statement (29).

### 2.1. Study design and population

National Health and Nutrition Examination Survey is a multistage, nationally representative cross-sectional study (28). The U.S. National Center for Health Statistics (NCHS) conducts a complex, stratified, multistage probability-cluster sampling design to select participants from the U.S. deinstitutionalized civilians (30, 31). Information was collected through in-home interviews and mobile examination center (MEC) visits and released by CDC in 2-year cycles from 1999 (32).

This study included participants in the NHANES from 1999 through 2018 who reported complete information on first-day total nutrient intakes and ASCVD diagnoses related questionnaire. We excluded participants under 20 years of age.

Trained interviewers use a computer-assisted personal interview (CAPI) system to ask for demographic information and characteristics during in-home interviews and MEC. Among them, age, gender, ethnicity, education, poverty, and smoking status were derived from demographic data. Body mass index (BMI) and blood pressure (BP) were extracted from examination data. Insurance information was from questionnaires in which people reporting medicare coverage were asked to show their Medicare cards.

### 2.2. Dietary inflammatory index

The DII was calculated from a 24-hour recall of dietary data on day one (16, 33, 34), including the types and amounts of foods and beverages consumed during the 24-hour period preceding the interview (midnight to midnight) collected in the MEC. Data are subsequently used to estimate intakes of energy, nutrients, and other food components from those foods and beverages validated by the Nutrition Methodology Working Group (35).

We calculate DII according to Hébert’s scheme (14), which evaluated the inflammatory effects of 45 nutrients and was widely used in assessing dietary inflammation (10, 14, 16, 26, 34). In this study, 28 of the 45 food parameters available in NHANES were used to calculate DII, including carbohydrates, protein, total fat, alcohol, fiber, cholesterol, saturated fat, MUFA, PUFA, n-3 fatty acids, n-6 fatty acids, niacin, vitamin A, thiamin (vitamin B1), riboflavin (vitamin B2), vitamin B6, vitamin B12, vitamin C, vitamin D, vitamin E, Fe, Mg, zinc, selenium, folic acid, beta-carotene, caffeine, and energy. Previous studies have shown that using fewer than 30 food parameters for DII calculations does not affect the predictive power of DII (16, 34, 36, 37). Data for the calculation of DII are presented in Supplementary Table 1. The calculation steps are as follows. Z score = (daily intake of a certain dietary ingredient or nutrient–global daily mean intake)/the standard deviation of the global mean per capita daily intake of this dietary ingredient or nutrient (14). Then converted the Z-score to a percentile scale, doubled the resulting percentile value, and subtracted “1” to achieve a symmetrical distribution centered on “0”. Finally, multiplied by the total inflammation score for each dietary component, the inflammatory index of each dietary ingredient or nutrient was calculated to obtain an individual DII (14, 33, 37). DII scores range from negative to positive, with lower scores indicating greater anti-inflammatory effects and higher scores implying stronger pro-inflammatory effects of the diet (14).

### 2.3. Outcome definitions

Our outcome is whether diagnosed with ASCVD. According to the *2013 ACC/AHA Guideline on the Treatment of Blood Cholesterol to Reduce Atherosclerotic Cardiovascular Risk in Adults* (9), ASCVD was defined as having at least one diagnosis of coronary heart disease, angina, heart attack, and stroke; hard criteria was defined as heart attack and stroke. We described the diagnosis methods based on NHANES in Supplementary Table 2.

### 2.4. Covariates

Covariates about individual characteristics included age, sex, race or ethnicity, BMI, education level, smoking status, poverty income ratio (PIR), and insurance. All detailed measurement procedures are available at www.cdc.gov/nchs/nhanes/publicly available. Physical activity data were analyzed according to the World Health Organization guidelines (38) and converted to metabolic equivalent (MET) minutes of moderate to vigorous physical activity per week (39). The diseases in the covariates included hypertension, hyperlipidemia, and diabetes, and their diagnosis basis was listed in Supplementary Table 2.

### 2.5. Statistical analysis

We performed analysis using appropriate NHANES sampling weights and took into account complex multistage cluster survey designs. The characteristics of participants were compared by chi-square test or *t*-test. The baseline information was presented as unweighted frequencies and weighted percentages for categorical variables; and for continuous variables, were shown as weighted mean and standard deviations (SD).

We constructed three models: Model 1 was the crude model without adjustment for any covariates. We adjusted age, sex, race or ethnicity, education level, smoking status, poverty, and insurance in addition to covariates in model 2. In model 3, we further adjusted BMI, hyperlipemia, hypertension, and diabetes in addition to covariates adjusted in model 2. The Independent variable DII was divided into ten deciles. The lowest 10% of DII score is deciles 1 (D1), followed by deciles 2 (D2), and the highest is deciles 10 (D10). Logistic regression was used to estimate the Odds Ratio (OR) and 95% confidence interval (95% CI) for ASCVD grouped by DII ranges. We further examined the non-linear relationship between DII and ASCVD using spline smoothing regression with the generalized additive model (GAM) (40, 41). Subsequently, we identified the inflection point by piecewise regression, which uses a separate line segment to fit each interval. A log-likelihood ratio test comparing the one-line model with the piecewise regression model was used to determine whether a threshold existed. We calculated OR through piecewise multivariable logistic regression if there was a threshold effect; otherwise, we directly used multivariable logistic regression. All analysis was based on weighted data.

In addition, we conducted a subgroup analysis stratified by the individual component of ASCVD (i.e., CHD, angina, heart attack, and stroke) and sex to assess whether there was a sex difference in the association of DII with ASCVD. The sensitivity analysis was conducted by using the second decile as the reference to perform the segmented linear regression of three models and further adjusted physical activity.

All analyses were conducted by R software (4.2.0) and Empower^2^ following CDC guidelines.^3^ *P*-values less than 0.05 were considered statistically significant.

## 3. Results

### 3.1. Characteristics of participants

**FIGURE 1:**
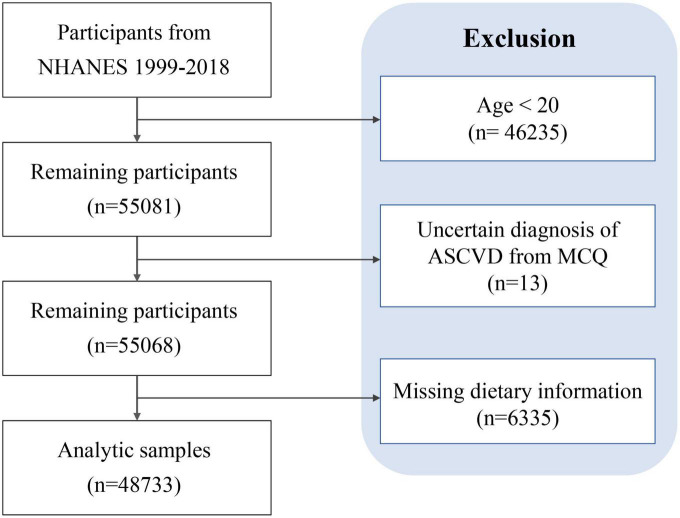
Flow chart of study participants.

A total of 48,733 participants were enrolled in this study after screening according to the established protocol, of which 5,011 (10.28%) participants were diagnosed with ASCVD (Figure 1 and Table 1). As shown in Table 1, the mean age was 47.13 ± 0.19 years old, and 51.91% were female. The mean DII score was 1.38 ± 0.02, ranging from −5.28 to 5.79. Compared with those not diagnosed with ASCVD, people diagnosed with ASCVD are more likely to be older, male, Non-Hispanic White, obese, less educated, former smokers, poverty, and have insurance.

**TABLE 1:** Baseline characteristics of participants, from National Health and Nutrition Examination Survey (NHANES) 1999–2018.

Characteristic	Overall	Non-ASCVD	Diagnosed ASCVD	*P*
***N***	48,733	43,722	5,011	
DII score, mean ± SD*	1.38 ± 0.02	1.36 ± 0.02	1.66 ± 0.05	<0.001
Age, mean ± SD*	47.13 ± 0.19	45.56 ± 0.18	64.66 ± 0.31	<0.001
**Age distribution, N (%)^#^**				<0.001
20–44 years	20,930 (46.76)	20,594 (50.15)	336 (8.93)	
45–64 years	15,825 (34.94)	14,245 (34.91)	1,580 (35.22)	
≥65 years	11,978 (18.30)	8,883 (14.94)	3,095 (55.85)	
**Sex, *N* (%)^#^**				<0.001
Female	25,231 (51.91)	23,115 (52.51)	2,116 (45.27)	
Male	23,502 (48.09)	20,607 (47.49)	2,895 (54.73)	
**Race or ethnic, *N* (%)^#^**				<0.001
Non-Hispanic White	21,864 (68.40)	19,035 (67.76)	2,829 (75.54)	
Non-Hispanic Black	10,195 (11.24)	9,180 (11.25)	1,015 (11.12)	
Mexican-American	8,554 (8.21)	7,965 (8.54)	589 (4.45)	
Other	8,120 (12.16)	7,542 (12.45)	578 (8.89)	
BMI, kg/m^2^, mean ± SD*	28.77 ± 0.07	28.66 ± 0.07	30.03 ± 0.13	<0.001
**BMI, kg/m^2^, N (%)^#^**				<0.001
Normal weight	13,369 (29.13)	12,335 (30.23)	1,034 (21.00)	
Low weight	755 (1.64)	691 (1.68)	64 (1.42)	
Overweight	16,233 (32.86)	14,576 (33.30)	1,657 (32.78)	
Obesity	17,618 (35.17)	15,564 (34.79)	2,054 (44.80)	
**Education level, *N* (%)^#^**				<0.001
<High school	13,045 (17.11)	11,266 (16.29)	1,779 (26.40)	
High school graduate	11,329 (24.17)	10,075 (23.81)	1,254 (28.32)	
Some college	13,810 (31.13)	12,528 (31.52)	1,282 (27.05)	
College graduate or above	10,489 (27.53)	9,803 (28.38)	686 (18.23)	
**Smoking status, *N* (%)^#^**				<0.001
Never smoked	26,393 (53.48)	24,466 (54.89)	1,927 (38.12)	
Former smoker	12,156 (24.90)	10,097 (23.61)	2,059 (39.45)	
Current smoker	10,148 (21.56)	9,125 (21.50)	1,023 (22.44)	
**Poverty income ratio, *N* (%)^#^**				<0.001
< = 1	9,194 (13.61)	8,134 (14.38)	1,060 (17.57)	
>1	35,413 (79.39)	31,884 (85.62)	3,529 (82.43)	
**Insurance, *N* (%)^#^**				<0.001
Any insurance	38,430 (82.03)	33,884 (81.51)	4,546 (91.12)	
No insurance	10,101 (17.63)	9,656 (18.49)	445 (8.88)	
**MET,** min/week, mean ± SD*	3,424.48 ± 62.75	3,463.99 ± 63.63	2,842.88 ± 137.27	<0.001
**Hyperlipidemia, *N* (%)^#^**				<0.001
No	14,692 (30.86)	13,975 (32.46)	717 (13.01)	
Yes	34,035 (69.12)	29,741 (67.54)	4,294 (86.99)	
**Hypertension, *N* (%)^#^**				<0.001
No	27,756 (61.79)	26,776 (65.29)	980 (22.75)	
Yes	20,977 (38.21)	16,946 (34.71)	4,031 (77.25)	
**Diabetes, *N* (%)^#^**				<0.001
No	35,661 (79.42)	33,015 (82.80)	2,646 (56.50)	
Diabetes	8,348 (12.79)	6,381 (10.93)	1,967 (35.51)	
Impaired fasting glucose	2,053 (3.97)	1,791 (3.89)	262 (5.64)	
Impaired glucose tolerance	1,270 (2.35)	1,140 (2.39)	130 (2.35)	
**CHD, *N* (%)^#^**				<0.001
No	46,474 (96.16)	43,620 (100.00)	2,854 (56.10)	
Yes	2,049 (3.55)	0 (0.00)	2,049 (43.90)	
**Angina, *N* (%)^#^**				<0.001
No	47,157 (97.24)	43,641 (100.00)	3,516 (68.57)	
Yes	1,415 (2.55)	0 (0.00)	1,415 (31.43)	
**Heart attack, *N* (%)^#^**				<0.001
No	46,478 (96.34)	43,682 (100.00)	2,796 (56.60)	
Yes	2,183 (3.55)	0 (0.00)	2,183 (43.40)	
**Stroke, *N* (%)^#^**				<0.001
No	46,775 (96.95)	43,684 (100.00)	3,091 (63.90)	
Yes	1,907 (2.97)	0 (0.00)	1,907 (36.10)	

^#^Mean ± SD: weighted mean and standard deviations (SD). *N (%): unweighted frequencies and weighted percentages.

### 3.2. Relationship between DII and ASCVD

We conducted a weighted multivariable logistic regression analysis to examine the association between DII and ASCVD (Table 2). In the crude model (model 1), participants in the five highest deciles of DII score (D6, 7, 8, 9, and 10) had a significantly higher risk of having ASCVD compared to those in decile 1 (D1). The adjustment for demo socioeconomic covariates (age, sex, race or ethnicity, education level, smoking status, poverty, and insurance) in model 2 attenuated the association [D9 vs. D1, OR = 1.30, 95% CI = (1.06,1.59); D10 vs. D1, OR = 1.67, 95% CI = (1.34,2.08)]. Additional adjustment for comorbidities (hyperlipemia, hypertension, and diabetes) and BMI in model 3 only slightly attenuated the association [D10 vs. D1, OR = 1.47, 95% CI = (1.18,1.84)].

**TABLE 2:** Association between dietary inflammatory index (DII) and atherosclerotic cardiovascular disease (ASCVD).

DII		Event/Total	OR (95% CI), weighted		
Decile	Range		Model 1^1^	Model 2^2^	Model 3^3^
D1	−5.28∼−1.14	400/4,874	1 (ref.)	1 (ref.)	1 (ref.)
D2	−1.14∼−0.14	419/4,873	1.04 (0.85,1.28)	1.00 (0.80,1.25)	0.92 (0.74,1.15)
D3	−0.14∼0.58	404/4,873	1.07 (0.88,1.29)	0.98 (0.79,1.20)	0.92 (0.75,1.13)
D4	0.58∼1.20	482/4,873	1.19 (0.98,1.45)	1.08 (0.86,1.35)	1.01 (0.81,1.26)
D5	1.20∼1.75	463/4,873	1.18 (0.98,1.43)	1.12 (0.92,1.38)	1.06 (0.86,1.30)
D6	1.75∼2.26	512/4,874	1.31 (1.07,1.61)*	1.20 (0.95,1.52)	1.12 (0.89,1.41)
D7	2.26∼2.72	499/4,873	1.31 (1.07,1.61)*	1.17 (0.94,1.46)	1.06 (0.85,1.33)
D8	2.72∼3.19	525/4,873	1.29 (1.05,1.57)*	1.08 (0.86,1.35)	0.97 (0.77,1.22)
D9	3.19∼3.73	588/4,873	1.50 (1.24,1.81)***	1.30 (1.06,1.59)*	1.17 (0.94,1.44)
D10	3.73∼5.79	719/4,874	1.87 (1.53,2.29)***	1.67 (1.34,2.08)***	1.47 (1.18,1.84)***

^1^Model 1 without adjustment for covariates. ^2^Model 2 adjusted age, sex, race or ethnicity, education level, smoking status, poverty, and insurance. ^3^Model 3 further adjusted body mass index, hyperlipemia, hypertension, and diabetes based on model 2. *P < 0.05, ***P < 0.001.

**FIGURE 2:**
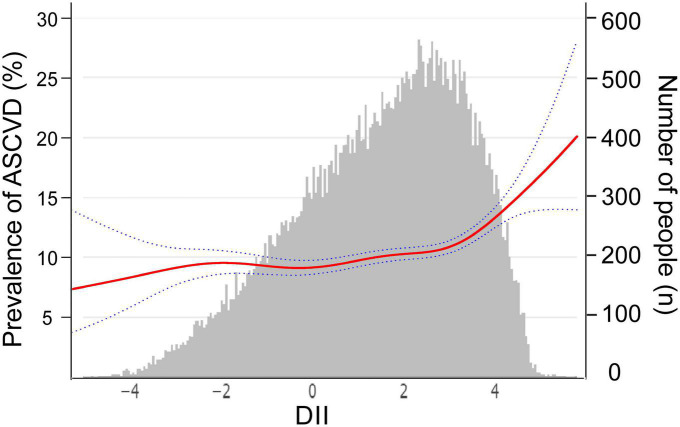
The relationship between dietary inflammatory index (DII) and atherosclerotic cardiovascular disease (ASCVD), weighted. The red curve represents the non-linear relationship between DII and ASCVD, and the dark blue dotted lines represent the 95% of confidence interval from the fit, corresponding to the vertical coordinate on the left. The gray histogram shows the DII distribution of participants, corresponding to the vertical coordinate on the right. We adjusted age, sex, race or ethnicity, body mass index, education level, smoking status, poverty, insurance, hyperlipemia, hypertension, and diabetes.

The above multivariable logistic regression suggests a non-linear relationship between DII and ASCVD, we thereby performed an adjusted spline smoothing regression to detect the dose-response and identify the cutoff point (Figure 2 and Supplementary Table 3). Figure 2 shows that the distribution of DII scores is skewed, and the curve representing the relationship between DII and ASCVD is divided into three segments, which the log-likelihood ratio test (Supplementary Table 3) also verified the existence of inflection points. Therefore, we divided the curve into three segments and further conducted a piecewise multivariable logistic regression analysis to explore the threshold effect (Table 3). As we can see, when DII is less than −2, although the curve shows an upward trend, there is no significant relationship between DII and ASCVD [OR = 0.86, 95% CI = (0.53,1.39), *P* = 0.53, *N* = 2,194]. There is a midly elevated trend between DII and ASCVD when DII ranges from −2 to 3 [OR = 1.05, 95% CI = (1.00,1.10), *P* = 0.04, *N* = 34,863]. Notably, when DII was greater than 3, the percentage of people diagnosed with ASCVD increased rapidly as DII increased [OR = 1.41, 95% CI = (1.15,1.73), *P* = 0.001, *N* = 11,676].

**TABLE 3:** Threshold effect analysis of the association between dietary inflammatory index (DII) and atherosclerotic cardiovascular disease (ASCVD), weighted.

DII range	N	OR (95% CI)	*P*
<−2	2,194	0.86 (0.53,1.39)	0.53
−2∼3	34,863	1.05 (1.00,1.10)	**0.04**
≥3	11,676	1.41 (1.15,1.73)	**0.001**

We adjusted age, sex, race or ethnicity, body mass index, education level, smoking status, poverty, insurance, hyperlipemia, hypertension, and diabetes. Bold represents *P* < 0.05.

### 3.3. Subgroup analysis

In the all-adjusted model, DII is not significantly associated with CHD, angina pectoris, or heart attack (Table 4, Supplementary Table 3, and Supplementary Figure 1); only for the outcome of stroke, when DII scores more than −1.5, each point related 11% [−1.5 ≤ DII < 3, OR = 1.11, 95% CI = (1.02,1.20), *P* = 0.01] or 44% [DII ≥ 3, OR = 1.44, 95% CI = (1.15,1.81)] increasing risk. The association between DII and hard criteria (i.e., heart attack and stroke) shows a similar trend to DII and ASCVD. There is no significant relationship between DII and hard criteria [OR = 0.81, 95% CI = (0.42,1.58), *P* = 0.53] when DII is less than −2. When DII ranges from −2 to 3, the prevalence of hard criteria increased slowly with DII [OR = 1.08, 95% CI = (1.03,1.14), *P* = 0.004]. Notably, the percentage of people diagnosed with a heart attack or stroke increased rapidly with increasing DII when DII was greater than 3 [OR = 1.28, 95% CI = (1.06,1.53), *P* = 0.01].

**TABLE 4:** Subgroup analysis of the association between dietary inflammatory index (DII) and atherosclerotic cardiovascular disease (ASCVD) stratified by sex and diseases.

Disease	Sex	DII	OR (95% CI)^#^	*P*
ASCVD				
	Female	continuous	1.13 (1.08,1.18)	**<0.001**
	Male	continuous	1.00 (0.96,1.04)	0.94
Hard criteria	Total	<−2	0.81 (0.42, 1.58)	0.53
		−2∼3	1.08 (1.03,1.14)	**0.004**
		≥3	1.28 (1.06,1.53)	**0.01**
	Female	continuous	1.13 (1.07,1.18)	**<0.001**
	Male	continuous	1.02 (0.97,1.07)	0.36
CHD	Total	continuous	1.01 (0.97,1.05)	0.58
	Female	continuous	1.08 (1.00,1.16)	0.05
	Male	continuous	0.98 (0.93,1.03)	0.46
Angina	Total	continuous	1.00 (0.95,1.06)	0.92
	Female	continuous	1.07 (1.00,1.14)	0.05
	Male	continuous	0.96 (0.89,1.03)	0.26
Heart attack	Total	continuous	1.04 (0.99,1.09)	0.12
	Female	continuous	1.12 (1.04,1.20)	**0.002**
	Male	<−1.5	0.64 (0.37, 1.10)	0.11
		−1.5∼0	0.63 (0.32,1.22)	0.17
		0∼2.5	1.03 (0.85,1.24)	0.77
		≥2.5	0.79 (0.60,1.03)	0.08
Stroke	Total	<−1.5	1.60 (0.90, 2.86)	0.11
		−1.5∼3	1.11 (1.02,1.20)	**0.01**
		≥3	1.44 (1.15,1.81)	**0.002**
	Female	<2.5	1.11 (0.98,1.25)	0.12
		≥2.5	1.25 (1.01,1.54)	**0.04**
	Male	<−2	3.95 (0.92,16.90)	0.06
		−2∼3.5	1.06 (0.97,1.15)	0.19
		≥3.5	2.29 (1.08,4.86)	**0.03**

^#^Weighted results. Hard criteria: including stroke and heart attack. We adjusted age, sex, race or ethnicity, body mass index, education level, smoking status, poverty, insurance, hyperlipemia, hypertension, and diabetes. Bold represents *P* < 0.05.

**FIGURE 3:**
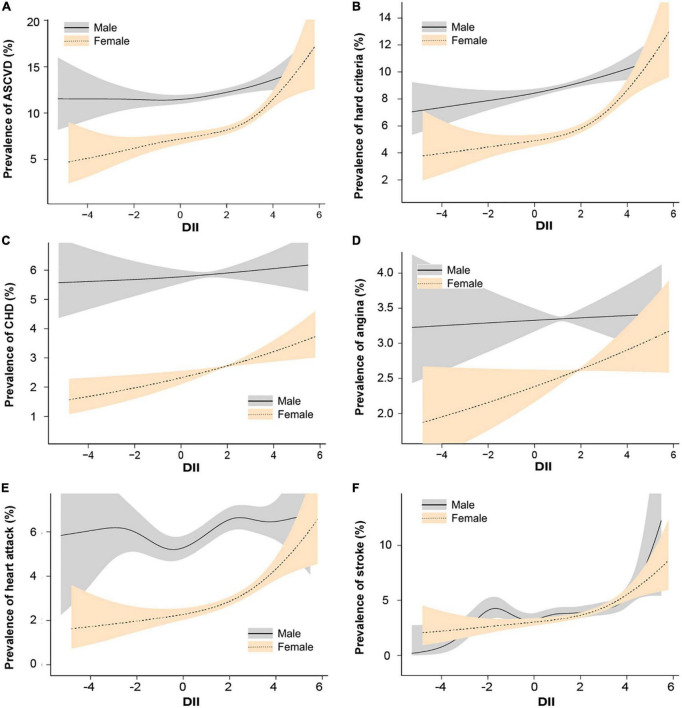
Spline smoothing of dietary inflammatory index (DII) and atherosclerotic cardiovascular diseases stratified by sex, weighted. We adjusted age, sex, race or ethnicity, body mass index, education level, smoking status, poverty, insurance, hyperlipemia, hypertension, and diabetes. **(A)** Atherosclerotic cardiovascular disease. **(B)** Hard criteria (including heart attack and stroke). **(C)** Coronary heart disease. **(D)** Angina. **(E)** Heart attack. **(F)** Stroke. The solid line represents males and the gray shading represents the 95% confidence interval for males. The need line represents females and the orange shading represents the 95% confidence interval for females.

We further conducted the smooth curve fittings of subgroup analysis stratification by atherosclerotic cardiovascular diseases and gender (Figure 3) and presented their linear or piecewise multivariable logistic regression (Table 4) according to the log-likelihood ratio (Supplementary Table 3). In the stratified analysis by sex (Figure 3A and Table 4), the overall prevalence of ASCVD in males was higher than that of females. In females, the probability of diagnosed ASCVD was proportional to DII [OR = 1.13, 95% CI = (1.08,1.18)]. While in males, there was no significant association between DII and ASCVD [OR = 1.00, 95% CI = (0.96,1.04)].

In other subgroups exploring the association with DII, female in heart attack [OR = 1.12, 95% CI = (1.04,1.20)] and stroke [DII ≥ 2.5, OR = 1.25, 95% CI = (1.01,1.54)] showed a significant relationship, while female in CHD [OR = 1.08, 95% CI = (1.00,1.16), *P* = 0.05] and angina [OR = 1.07, 95% CI = (1.00,1.14), *P* = 0.05] only shows a borderline significant.

### 3.4. Sensitivity analysis

Given the large confidence interval of the first decile, we used the second decile as the reference for segmented linear regression (Supplementary Table 4). The sensitivity analysis supports the above results. Furthermore, the multiple logistic regressions with or without adjusting physical activity between DII and ASCVD (Supplementary Table 5) and in subgroup analysis (Supplementary Table 6) are consistent.

## 4. Discussion

An unhealthy diet is a well-established risk factor for ASCVD (42), one of the fundamental mechanisms is that an unhealthy diet contributes to chronic low-grade inflammation in the gut and the whole body (43). Using a nationally representative cross-sectional study of U.S. adults, the current research involving 48,733 participants demonstrated a significant association between DII and ASCVD. Notably, we observed a non-linear association between DII and ASCVD, with an abruptly increased risk of having ASCVD when the DII score is higher than 3. Additionally, we found that the association of DII with ASCVD was stronger in women than in men.

Studies on DII began in 2004 (44). In 2009, Hébert et al. formulated the first version of DII to provide a pooled measure of diet-related inflammation (13) and revised it in 2014 (14). Subsequently, multiple studies validated the ability of DII in predicting chronic inflammatory disease (10, 45). The DII is universally applicable because it involves the six most commonly inflammatory markers [interleukin(IL)-1β, IL-4, IL-6, IL-10, tumor necrosis factor (TNF)-α, and C-reactive protein] (44). We can calculate the DII scores from any dietary assessment tool based on nutrient intake data (44). Furthermore, the DII score is positively related to the glycemic index (GI) (46) and negatively correlated with the Healthy Eating Index-2010 (HEI-2010), the Alternative Healthy Eating Index (AHEI), and the Dietary Approaches to Stop Hypertension Index (DASH) (21, 47–49). That is, an anti-inflammatory diet is healthier than a pro-inflammatory diet evaluated by other diet indexes, such as HEI-2010, AHEI, DASH, and GI scores, which are consistent with the meaning of the DII score. Besides, DII has the added advantage of quantifying the diet’s inflammatory potential.

Our finding that higher DII was associated with an increased risk of having ASCVD was consistent with previous studies. A meta-analysis (27), including 14 observational studies, suggested that the highest versus lowest DII quartile was associated with a 35% [OR = 1.35, 95% CI = (1.11, 1.63), I^2^ = 36%] increase in CVD risk, and for each one-point increase in the DII score, the risk increased by 8% [OR = 1.08, 95% CI = (1.00, 1.16), I^2^ = 71%]. The SUN (Seguimiento Universidad de Navarra) Cohort (15) enrolling 18,794 Spanish university graduates and with 8.9 years of follow-up, found that participants in the highest quartile of DII scores had approximately twice the risk of CVD compared with the lowest quartile [hazard ratio (HR) = 2.03, 95% CI = (1.06–3.88), *N* = 18,794].

Contrary to our findings, an Australian women cohort indicated no association between DII and risk of total CVD, ischemic heart disease, myocardial infarction, cerebrovascular disease, or stroke (50). Another sub-analysis of the MASHAD (Mashhad stroke and heart atherosclerotic disorder) cohort study also found no statistically significant association between the DII and total CVD, myocardium infarction (MI), stable angina (SA), or unstable angina (UA) in middle-aged Iranians (51). However, this study included 4,672 participants, of whom only 124 developed CVD; the sample size might influence the results. Moreover, Catherine et al. (10) explained that race is one of the factors affecting the relationship between DII and CVD; it is more potent in Europe, North America, and Japan than in Australia and Iran.

Notably, our study goes beyond the previous reports (52) by finding a non-linear relationship between DII and ASCVD incidence. When DII was less than −2, no significant association between DII and ASCVD was observed; However, when DII was 3 or greater, one increase of DII was associated with a 41% increase in ASCVD prevalence. This non-linear association observed in the present study indicated that a certain amount of food or elements with pro-inflammatory properties such as vitamin B12, energy, protein, and cholesterol, would not increase the risk of ASCVD probably due to the fact that these elements are essential for basic life activities (14). Only an excessive amount of food or elements with pro-inflammatory properties much greater than needed would increase the risk of ASCVD. This threshold effect has been observed in the relationship between DII score and serum Klotho (23), sex hormones (24), and depression (25).

The potential mechanism of the observed association between DII and ASCVD could be explained by inflammation. Accumulating evidence suggests that ASCVD is a chronic inflammatory disease (53). Shivappa et al. (54) analyzed 532 participants in the HELENA-CSS (Healthy Lifestyle in Europe by Nutrition in Adolescence Cross-Sectional Study) and found that a diet with higher DII scores was related to increased levels of TNF-α, IL-1, IL-2, IFN-γ, and vascular cell adhesion molecule. In the AUSMED heart trial (AUStralian MEDiterranean Diet Heart Trial) (55), after 6 months of dietary intervention, improvement in DII scores was associated with a reduction in IL-6 among Australians diagnosed with CHD (*n* = 65). Juliana et al. (56) included 329 adolescents from the LabMed Physical Activity Study, and reported that the DII score is positively correlated with component C4 and IL-6. The increased levels of inflammatory cytokines may subsequently induce the migration of inflammatory cells into vascular tissues or mediate leukocyte adhesion to the vascular endothelium by increasing the expression of cell adhesion molecules (10, 57, 58).

Our analysis indicated DII was associated with stroke but irrelevant to CHD, angina, or heart attack in the adjusted model. A previous study conducted by Wirth et al. among U.S. adults (52) partly supported our findings. They found that compared to participants in the lowest quartile of DII, those in DII the highest quartile were more likely to have a stroke and heart attack, but no significant relevance was observed for CHD or angina (52). However, this study does not adjust for hyperlipemia, hypertension, or diabetes, which may lead to biased results because hyperlipemia, hypertension, and diabetes are independent risk factors for ASCVD (42) and could be influenced by a pro-inflammation diet (16, 18, 52). In line with our result, several studies have shown a positive association between DII and stroke in Korean (59), Japanese (60), and U.S. (52), while studies in French (61) and Australian women (50) show no relevance. The association between DII and MI risk is also disputable; studies in Iran (51), Australian women (50), and Korean (59) are consistent with ours, while the studies among French (61) and Italian (62) reported a positive association between DII and MI (61, 62). A possible explanation for this could be that the underlying mechanisms of inflammation and its effects on subtypes of ASCVD vary by sex and country (43, 59, 63, 64). In addition, in NHANES survey, the diagnosed of angina and CHD was according to questionnaire and some case without further details. The inaccurate diagnosis may also account for the negative results in the CHD and angina subgroups in the current study. Furthermore, the subgroup of hard criteria including heart attack and stroke shows a similar trend compared with ASCVD, which supports the above speculation.

Recently, Rohit et al. reviewed the literature on DII studies and pointed out that the association between ASCVD and DII is potentially sex-specific (43). Since 1984, the annual CVD mortality rate has remained greater for women than for men, and the absolute numbers of individuals living with and dying of CVD in the United States are larger for women than for men (65). In line with previous studies, our results show that sex (stronger in females than males) seems to be the key factor affecting the relationship between DII and ASCVD (10, 52). In females, elevated DII was significantly associated with the prevalence of ASCVD, heart attack, and stroke, and there was a borderline significant association with the prevalence of CHD and angina. In males, however, only in stroke were significant. Contrary to our findings, the KoGES_HEXA (Korean Genome and Epidemiology Study Health Examination) cohort showed that a pro-inflammatory diet significantly increased the risk of CVD in males, while there was no significant in females (59). In another Korean survey, the relation between DII and the 10-Year Risk for ASCVD also found that male in the highest quartile has a greater risk than those in the lowest quartile (HR = 1.34, *N* = 4,185), while there is no significant difference in female (26). We can hypothesize that inflammation has a differential effect in predicting chronic disease outcomes between sexes in agreement with the current literature. A cohort study using data from the Multiethnic Cohort Study in Hawaii and California, demonstrated that for the highest vs. lowest quintile of the DII in men and women were 1.15 [95% CI = (1.09,1.21)] and 1.22 [95% CI = (1.14,1.28)] for all-cause mortality, 1.13 [95% CI = (1.03,1.23)] and 1.29 [95% CI = (1.17,1.42)] for CVD, and 1.10 [95% CI = (1.00,1.21)] and 1.13 [95% CI = (1.02,1.26)] for cancer mortality, which supported a stronger association in women (66). In a recent meta-analysis, an increased risk of CVD (either risk of incident disease or mortality) with higher DII scores was significant only in women and in studies conducted in Europe and North America, but not in men and studies conducted in Australia (27). In other chronic disease studies, both the Women’s Health Initiative and the Iowa Women’s Health Study found that women who consumed the most pro-inflammatory diets had an 20% increased risk of colorectal cancer compared with women who consumed the most anti-inflammatory diets (67, 68). A case-control study conducted in Korea showed that higher DII scores were associated with an increased incidence of colorectal cancer. This association was stronger in women [OR = 2.50, 95% CI = (1.64, 3.82)] than men [OR = 1.72, 95% CI = (1.30, 2.28)] (69). The reasons for the inconsistent results could be as follows: First, it has been discussed above that nationality is an essential factor influencing the relationship between DII and ASCVD. Secondly, in our study, the prevalence of various atherosclerotic diseases was higher in males than in females in almost all DII ranges. Vulnerable plaque rupture and thrombosis are the leading causes of stroke and MI (70). Plaques from females tend to be more stable (71), while those from males are more inflamed and have additional unstable features (72, 73). Third, this may also be indirectly due to sex differences in CRP, GI, and sex hormones in DII. However, the specific mechanism is still unknown to our knowledge, which may be a promising direction for the further research.

There are several studies performed in women versus men of DII versus GI, CRP, and sex hormones. Intervention studies showed that a low GI diet (74, 75) lowered plasma CRP in short-term and long-term studies in overweight and obese adults. High GI foods, which are characteristically highly refined carbohydrates and/or carbohydrates with little fiber, are one of the major dietary factors affecting inflammation. However, the relationship between inflammation and GI is more notable in females. A cross-sectional study reported that a strong positive relationship between dietary GI (76) and plasma CRP in healthy, middle-aged women. In another cross-sectional study (46), increased inflammatory potential of diet, as represented by higher DII scores, was associated with increased GI scores. Moreover, the correlation coefficient between DII score and GI score was higher in women than in men (0.32 vs. 0.27), implying that the association of DII with GI was stronger in women. The DII has been positively associated with inflammatory biomarkers such as CRP (i.e., a pro-inflammatory diet leads to a higher inflammation) (77, 78). Furthermore, the study shows that the DII was significantly associated with inflammatory biomarkers in post-menopausal women (79). A cross-sectional study using data of the National Integrated Project for Prospective Observation of Non-communicable Disease and its Trends in the Aged 2010, indicated that DII score was positively associated with hs-CRP. However, this relationship was only stable among women (Men: standardized β = 0.05, *P* = 0.14; Women: standardized β = 0.06, *P* = 0.02) (80), which was consistent with our findings. Sex hormones and sex hormone binding globulin (SHBG) are easily affected by inflammation. A cross-sectional study included 2,092 female participants (age ≥ 20) from the 2013–2016 National Health and Nutrition Examination Survey demonstrated that a pro-inflammatory diet caused decreased SHBG in adult women (34). Karelis et al. found that vegetarians presented higher concentrations of SHBG in both pre- and post-menopausal women due to higher levels of fiber intake (81). Another cross-sectional study showed that energy-adjusted DII was positively associated with testosterone (*P* = 0.035), free testosterone (*P* = 0.026) and testosterone/estradiol (*P* = 0.065) in post-menopausal women (24). Thus, the association between DII and sex hormones is apparent in women, and no studies have explored this relationship in adult men, possibly due to the fact that hormonal changes in women are more affected by inflammation.

Our study had several strengths. First, to our knowledge, this is the first study to explore the relationship between DII and ASCVD in U.S. adults. The large sample size and complex sampling from the general population make it possible to extrapolate to the U.S. population. Second, compared with previous studies, we additionally explored the non-linear relation of DII with ASCVD and the potential cutoff point (2.2) for the relation. Third, the curve fitting and segmented linear regression results were consistent, indicating stable and reliable results. Finally, our subgroup analysis identified the populations and diseases affected most by DII. Overall, the current CVD guidelines have not considered DII as a diet recommendation (42). We demonstrated that higher DII was associated with an increased risk of ASCVD, which provides a reference and cutoff value for future research and diet recommendations.

In interpreting these results, several limitations should be taken into account. First, the cross-sectional study can only examine correlations but difficult to make a causal inference or investigate the temporal relationships (82). Subsequent large cohort studies or randomized controlled trials are necessary to confirm the findings. Second, ASCVD was defined based on diagnostic information, which may miss patients unaware of their disease. The diagnosed of CHD and angina was according to questionnaire and some case without further details, which may lead to an inaccurate diagnosis. In addition, due to the limited information collection in NHANES, the current study was unable to include peripheral vascular diseases in ASCVD. These may lead to bias. Further studies should consider the spectrum of diseases caused by atherosclerosis to clarify the relationship between DII and ASCVD further. Third, the DII was calculated based on a single 24-hour dietary recall, which is subject to some chance and recall bias. Nevertheless, this method is currently widely used and validated (16, 33, 34). Forth, due to the limited information in the NHANES database, carbohydrates were jointly taken into account without separating the complex from simple carbohydrates in calculating the DII score (83, 84). However, this study accounts for fiber, a good summary measure for complex carbohydrates (20). Perhaps the next generation of DII calculations could consider separating simple and complex carbohydrates. That is necessary, especially in women whose BMI might be more sensitive than in men to the carbohydrates’ components or ratio.

## 5. Conclusion

DII was positively and non-linearly associated with ASCVD in U.S. adults. This relationship was more pronounced in females. The findings could provide a reference for future research hypotheses and need to be further studied.

## Data availability statement

The original contributions presented in this study are included in the article/Supplementary material, further inquiries can be directed to the corresponding authors.

## Ethics statement

The studies involving human participants were reviewed and approved by the NCHS Research Ethics Review Board. Written informed consent for participation was not required for this study in accordance with the national legislation and the institutional requirements.

## Author contributions

HX, JQJ, JL, and JZ designed this study. RL and XC cleaned the data. JZ and JJ performed the analysis and revised the draft. WT normalized the pictures. XW and QL re-checked the data. JZ wrote the original draft. HX, JQJ, and JL reviewed the manuscript. All authors contributed to the article and approved the submitted version.

## Footnotes

1
https://www.cdc.gov/nchs/nhanes/index.htm

2
http://www.empowerstats.net/analysis/

3
www.cdc.gov/nchs/nhanes/
