# Supplementary material for: Association between dietary inflammatory index and atherosclerosis cardiovascular disease in U.S. adults
Source: Front Nutr. 2023 Jan 5;9:1044329. doi: 10.3389/fnut.2022.1044329 (PMC9849765; doi:10.3389/fnut.2022.1044329)
Supplement: Supplementary file 1 [file Data_Sheet_1.docx]

Supplementary Material

Supplementary Table 1. The food parameters used to calculate DII in this study.

Supplementary Table 2. Disease diagnosis from NHANES database.

Supplementary Table 3. Log-likelihood ratio of the spline smoothing.

Supplementary Figure 1. Graphics of smooth curve fittings of DII and atherosclerotic cardiovascular diseases, weighted.

Supplementary Table 4. Logistics regression between ASCVD and DII, weighted.

Supplementary Table 5. Association between DII and ASCVD, weighted.

Supplementary Table 6. Subgroup analysis of the association between DII and ASCVD, weighted.

# Supplementary Table 1. The food parameters used to calculate DII in this study.

| **Food parameters** | **Raw inflammatory effect score** | **Overall inflammatory effect score** | **Global daily mean intake (units/d)** | **SD** |
| --- | --- | --- | --- | --- |
| Alcohol | -0.278 | -0.278 | 13.98 | 3.72 |
| Beta-carotene | -0.584 | -0.584 | 3718 | 1720 |
| Caffeine | -0.124 | -0.11 | 8.05 | 6.67 |
| Carbohydrates | 0.109 | 0.097 | 272.2 | 40 |
| Cholesterol | 0.347 | 0.11 | 279.4 | 51.2 |
| Energy | 0.18 | 0.18 | 2056 | 338 |
| Fe | 0.032 | 0.032 | 13.35 | 3.71 |
| Fiber | -0.663 | -0.663 | 18.8 | 4.9 |
| Folic acid | -0.207 | -0.19 | 273 | 70.7 |
| Mg | -0.484 | -0.484 | 310.1 | 139.4 |
| MUFA | -0.019 | -0.009 | 27 | 6.1 |
| n-3 fatty acids | -0.436 | -0.436 | 1.06 | 1.06 |
| n-6 fatty acids | -0.159 | -0.159 | 10.8 | 7.5 |
| Niacin | -1 | -0.246 | 25.9 | 11.77 |
| Protein | 0.049 | 0.021 | 79.4 | 13.9 |
| PUFA | -0.337 | -0.337 | 13.88 | 3.76 |
| Riboflavin (vitamin B2) | -0.727 | -0.068 | 1.7 | 0.79 |
| Saturated fat | 0.429 | 0.373 | 28.6 | 8 |
| Selenium | -0.191 | -0.191 | 67 | 25.1 |
| Thiamin (vitamin B1) | -0.354 | -0.098 | 1.7 | 0.66 |
| Total fat | 0.298 | 0.298 | 71.4 | 19.4 |
| Vitamin B12 | 0.205 | 0.106 | 5.15 | 2.7 |
| Vitamin B6 | -0.379 | -0.365 | 1.47 | 0.74 |
| Vitamin A | -0.401 | -0.401 | 983.9 | 518.6 |
| Vitamin C | -0.424 | -0.424 | 118.2 | 43.46 |
| Vitamin D | -0.446 | -0.446 | 6.26 | 2.21 |
| Vitamin E | -0.419 | -0.419 | 8.73 | 1.49 |
| Zinc | -0.313 | -0.313 | 9.84 | 2.19 |

Data was selected from https://www.cambridge.org/core/journals/public-health-nutrition/article/designing-and-developing-a-literaturederived-populationbased-dietary-inflammatory-index/30BE2C2295CE93DC6B54F9F9AD50CC68.

**Reference:** Shivappa N, Steck S E, Hurley T G, et al. Designing and developing a literature-derived, population-based dietary inflammatory index[J]. Public Health Nutr, 2014, 17(8): 1689-96.

# Supplementary Table 2. Disease diagnosis from NHANES database.

| **Disease** | **Section** | **English Text** | **Value Description for Diagnosis Yes** |
| --- | --- | --- | --- |
| angina | Questionnaire Data | Has a doctor or other health professional ever told {you/SP} that {you/s/he} . . .had angina (an-gi-na), also called angina pectoris? | Yes |
| heart attack | Questionnaire Data | Has a doctor or other health professional ever told {you/SP} that {you/s/he} . . .had a heart attack (also called myocardial infarction (my-o-car-dee-al in-fark-shun))? | Yes |
|  | Questionnaire Data | Has a doctor or other health professional ever told {you/SP} that {you/he/she} had …? | a heart attack? |
| stroke | Questionnaire Data | Has a doctor or other health professional ever told {you/SP} that {you/s/he} . . .had a stroke? | Yes |
|  | Questionnaire Data | Has a doctor or other health professional ever told {you/SP}that {you/he/she} had . . .? | a stroke? |
| coronary heart disease | Questionnaire Data | Has a doctor or other health professional ever told {you/SP} that {you/s/he} . . .had coronary (kor-o-nare-ee) heart disease? | Yes |
| diabetes | Questionnaire Data | The next questions are about specific medical conditions. {Other than during pregnancy, {have you/has SP}/ {Have you/Has SP}} ever been told by a doctor or health professional that {you have/{he/she/SP} has} diabetes or sugar diabetes? | Yes |
|  | Laboratory Data | Glycohemoglobin | > 6.5% |
|  | Laboratory Data | Fasting glucose | ≥7.0 mmol/L |
|  | Laboratory Data | random blood glucose | ≥11.1 mmol/L |
|  | Laboratory Data | Two Hour Glucose (OGTT) | ≥11.1 mmol/L |
|  | Questionnaire Data | Use of diabetes medication or insulin, including biguanides (metformin), sulfonylureas, insulin, thiazolidinediones, dipeptidyl peptidase 4 inhibitors, glucose like peptide-1 receptor agonists, sodium-glucose co-transporter-2 inhibitors, alpha-glucosidase inhibitors, meglitinides, amylin analogs, other antidiabetic agents. |  |
| hyperlipidemia | Questionnaire Data |  |  |
|  | Laboratory Data | TG | ≥150 mg/dL |
|  | Laboratory Data | TC | ≥200 mg/dL |
|  | Laboratory Data | LDL-C | ≥130 mg/dL |
|  | Laboratory Data | HDL-C | ≤40mg/dL (male), 50mg/dL (female) |
|  | Questionnaire Data | Use of cholesterol-lowering drugs, including statins, including statins, ezetimibe, niacin and its derivatives, phenoxyaryl acids and their derivatives. |  |
| hypertension | Questionnaire Data | {Were you/Was SP} told on 2 or more different visits that {you/s/he} had hypertension, also called high blood pressure? | Yes |
|  | Questionnaire Data | {Have you/Has SP} ever been told by a doctor or other health professional that {you/s/he} had hypertension, also called high blood pressure? | Yes |
|  | Examination Data | Average SBP, average DBP (at least 3 times) | Average SBP≥140 mmHg, average DBP≥90 mmHg |
|  | Questionnaire Data | Use of antihypertensive medication, including angiotensin converting enzyme inhibitors, angiotensin receptor blockers, diuretics, calcium channel blockers, beta blockers, alpha blockers, centrally acting agents, direct vasodilators, aldosterone receptor antagonists, renin inhibitors, other antihypertensive agents. |  |

# Supplementary Table 3. Log-likelihood ratio of the spline smoothing.

| **Disease** | Log-likelihood ratio | | |
| --- | --- | --- | --- |
|  | Total | Female | Male |
| ASCVD | <0.001 | 1.000 | 1.000 |
| Hard criteria | <0.001 | 1.000 | 1.000 |
| CHD | 1.000 | 1.000 | 1.000 |
| Angina | 1.000 | 1.000 | 1.000 |
| Heart attack | 1.000 | 1.000 | <0.001 |
| Stroke | <0.001 | <0.001 | <0.001 |

Hard criteria: including stroke and heart attack.

# Supplementary Figure 1. Graphics of smooth curve fittings of DII and atherosclerotic cardiovascular diseases, weighted.


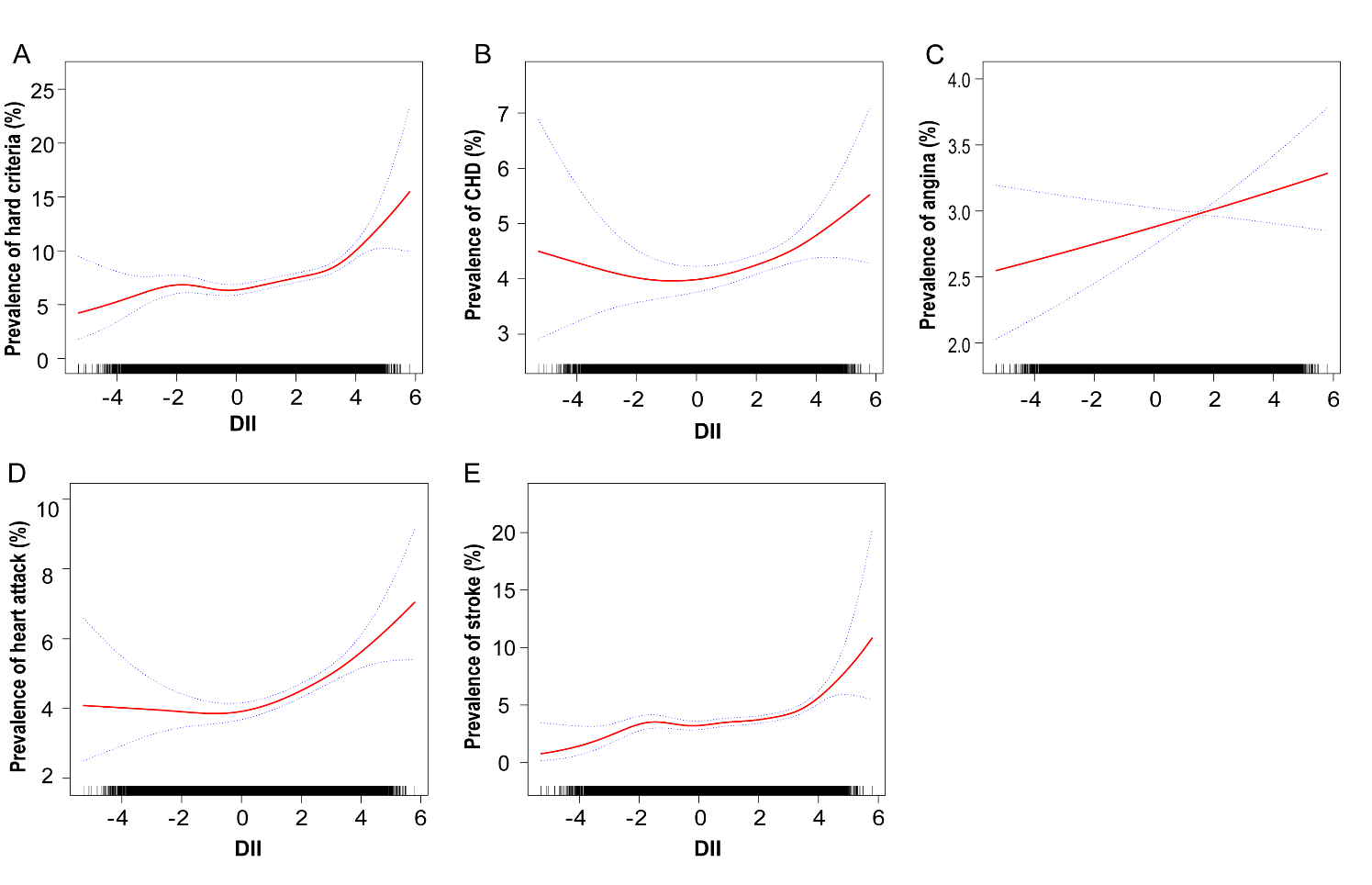


The red line represents the relationship between DII and **(A)** hard criteria (i.e., heart attack and stroke), **(B)** coronary heart disease, **(C)** angina, **(D)** heart attack, and **(E)** stroke. The dark blue dashed line represents the 95% of confidence interval from the fit. Adjustment factors included age, sex, race or ethnicity, weight status, education level, smoking status, poverty, insurance, hyperlipemia, hypertension, and diabetes.

**Supplementary Table 4. Logistics regression between ASCVD and DII, weighted.**

| **DII** | | **Odds Ratio (95% CI), weighted** | | |
| --- | --- | --- | --- | --- |
| **Decile** | **Range** | **Model 1**^1^ | **Model 2**^2^ | **Model 3**^3^ |
| **D1** | -5.28~ -1.14 | 0.96 (0.78,1.18) | 1.00 (0.80,1.25) | 1.09 (0.87,1.36) |
| **D2** | -1.14~ -0.14 | 1 (ref.) | 1 (ref.) | 1 (ref.) |
| **D3** | -0.14~ 0.58 | 1.02 (0.85,1.24) | 0.97 (0.79,1.21) | 1.00 (0.81,1.25) |
| **D4** | 0.58~ 1.20 | 1.14 (0.95,1.37) | 1.08 (0.87,1.33) | 1.10 (0.88,1.36) |
| **D5** | 1.20~ 1.75 | 1.14 (0.94,1.37) | 1.12 (0.91,1.38) | 1.15 (0.93,1.43) |
| **D6** | 1.75~ 2.26 | 1.26 (1.01,1.57) ^*^ | 1.20 (0.93,1.54) | 1.22 (0.94,1.58) |
| **D7** | 2.26~ 2.72 | 1.26 (1.04,1.51) ^*^ | 1.17 (0.96,1.42) | 1.16 (0.95,1.41) |
| **D8** | 2.72~3.19 | 1.24 (1.02,1.49) ^*^ | 1.07 (0.87,1.33) ^*^ | 1.06 (0.85,1.31) |
| **D9** | 3.19~ 3.73 | 1.44 (1.18,1.76) ^***^ | 1.29 (1.03,1.62) ^***^ | 1.27 (1.01,1.60) ^*^ |
| **D10** | 3.73~ 5.79 | 1.80 (1.51,2.15) ^***^ | 1.66 (1.36,2.04) ^***^ | 1.60 (1.30,1.97) ^***^ |

^1^ Model 1 without adjustment for covariates. ^2^ Model 2 adjusted age, sex, race or ethnicity, education level, smoking status, poverty, and insurance. ^3^ Model 3 further adjusted body mass index, hyperlipemia, hypertension, and diabetes based on model 2. **P*<0.05, ***P*<0.01, ****P*<0.001.

**Supplementary Table 5. Association between DII and ASCVD, weighted.**

| **Decile** | **DII Range** | **OR (95% CI)** | ***P*** |
| --- | --- | --- | --- |
| **D1** | -5.28~ -1.14 | 1 (ref.) |  |
| **D2** | -1.14~ -0.14 | 1.01 (0.78,1.31) | 0.95 |
| **D3** | -0.14~ 0.58 | 0.94 (0.74,1.20) | 0.63 |
| **D4** | 0.58~ 1.20 | 1.05 (0.80,1.37) | 0.72 |
| **D5** | 1.20~ 1.75 | 1.12 (0.86,1.47) | 0.39 |
| **D6** | 1.75~ 2.26 | 1.12 (0.83,1.51) | 0.47 |
| **D7** | 2.26~ 2.72 | 1.07 (0.80,1.43) | 0.64 |
| **D8** | 2.72~3.19 | 0.97 (0.72,1.32) | 0.87 |
| **D9** | 3.19~ 3.73 | 1.14 (0.88,1.47) | 0.32 |
| **D10** | 3.73~ 5.79 | 1.51 (1.15,1.99) | **0.004** |

Hard criteria: including stroke and heart attack. We adjusted age, sex, race or ethnicity, body mass index, education level, smoking status, poverty, insurance, physical activity, hyperlipemia, hypertension, and diabetes. Bold represents *P* < 0.05.

**Supplementary Table 6. Subgroup analysis of the association between DII and ASCVD, weighted.**

| **Disease** | **Sex** | **DII** | **OR (95% CI)** | ***P*** |
| --- | --- | --- | --- | --- |
| ASCVD | Total | < 3 | 1.02 (0.98,1.08) | 0.33 |
|  |  | ≥ 3 | 1.53 (1.16,2.03) | **0.003** |
|  | Female | continuous | 1.09 (1.03,1.15) | **0.003** |
|  | Male | continuous | 1.01 (0.96,1.05) | 0.80 |
| Hard criteria | Total | continuous | 1.05 (1.01,1.09) | **0.03** |
|  | Female | continuous | 1.09 (1.03,1.16) | **0.01** |
|  | Male | continuous | 1.01 (0.96,1.07) | 0.71 |

Hard criteria: including stroke and heart attack. We adjusted age, sex, race or ethnicity, body mass index, education level, smoking status, poverty, insurance, physical activity, hyperlipemia, hypertension, and diabetes. Bold represents *P* < 0.05.
